# Supplementary material for: PHYLOSCANNER: Inferring Transmission from Within- and Between-Host Pathogen Genetic Diversity
Source: Mol Biol Evol. 2017 Nov 23;35(3):719–33. doi: 10.1093/molbev/msx304 (PMC5850600; doi:10.1093/molbev/msx304)
Supplement: Supplementary Data [file msx304_supp.pdf]

PHYLOSCANNER: Inferring Transmission from Within- and Between-Host Pathogen Genetic Diversity

Chris Wymant, Matthew Hall, Oliver Ratmann, David Bonsall, Tanya Golubchik, Mariateresa de Cesare, Astrid Gall, Marion Cornelissen, Christophe Fraser, The Stop-HCV Consortium, The Maela Pneumococcal Collaboration, and The BEEHIVE Collaboration

Supplementary Information

SI 1: Ancestral state reconstruction

Throughout this section, we use "host" to refer to the individual experiencing infection or colonisation by a pathogen lineage; this will often be a patient experiencing clinical illness but may be an asymptomatic carrier or even, in a study of a disease of agriculture, a location. We use parsimony to perform an ancestral state reconstruction to internal nodes of a fixed phylogeny, where the states are the sampled hosts and a single extra "unassigned" state. The unassigned state is for lineages that must have infected hosts outside the dataset, and also regions where this cannot be ruled out by examination of the topology alone. This allows us to partially estimate the transmission process by identifying regions in the phylogeny where the reconstructed host changes.

Phylogenies taken as input to these reconstruction processes are built from a set of sequences, the majority of which will be obtained from samples isolated from a study population of interest. However, not every sequence need come from that population. Some may be reference isolates, and the user may wish to exclude others from consideration when reconstructing the transmission process as likely contaminants; indeed `phyloscanner` itself contains a tool to do just this. While such contaminant tips would ideally be excluded by repeating the phylogenetic inference with those sequences removed, for large datasets rebuilding the tree may be prohibitive in terms of time, and `phyloscanner` can instead simply be told to ignore or "blacklist" these tips when performing the reconstruction.

Trees are assumed to be rooted, but not necessarily bifurcating. Zero-length internal branches in the output of phylogenetics packages should be collapsed to form single, multifurcating nodes; this can be done as part of the package.

Because of differing substitution rates across the genome, branch lengths in different genomic windows can be quite variable. As these are used as a measure of genetic distance between hosts in the study population in what follows, and it is preferable that these distances not vary by position in the genome, `phyloscanner` offers the option to normalise branch lengths in every window tree. For our analysis of HIV-1 data we created a distance normalisition over the genome as follows. Starting with the '2015 Compendium: All M group' alignment of standard whole-genome reference sequences from the Los Alamos National Laboratory HIV database, we created sub-alignments in sliding windows along the genome, each containing 301 bp of the HXB2 sequence (and more or less of other sequences in proportion to their indels with respect to HXB2). Each window started 1bp after the previous one started, so that two consecutive sub-alignments share 300 of their 301 bp. For each window a maximum-likelihood phylogeny was inferred with `RAxML`. In each phylogeny, artifactually long tip branches were diagnosed with a Grubbs outlier test and p-value threshold 0.01, and removed; we then took the median of the distribution of all possible pairwise

patristic distances to characterise branch length in this window. To obtain a per-site measure this from per-window measure, for each site the mean of all windows spanning the site was taken.

SI 1.1 Romero-Severson-like reconstruction

Romero-Severson *et al.* (PNAS 2016) used an algorithm for the annotation of internal nodes with hosts that is equivalent to a maximum parsimony reconstruction when only two hosts are involved. In the more general case it is not, and lacks full mathematical rigour, but it often produces similar results and has the advantage of being very fast. First, each non-blacklisted tip is given a state corresponding to the host the corresponding sequence was sampled from, and every blacklisted tip a character "*".

The algorithm proceeds by performing a post-order traversal of the tree, at each node reconstructing a host state if that state is shared by the majority of its child nodes which were not given "*". If two or more hosts states are tied for the majority, then the node is instead given "*". Because this procedure can reconstruct host states deep into the tree (towards the root) if they happen to only encounter "*"s on the way, we also insist that reconstructions of a given host state are not allowed for any nodes ancestral to the most recent common ancestor (MRCA) node of the tips taken from that host; if that would happen, "*" is placed instead.

At the end of the process, any nodes given "*" that lie on an ancestral path between two nodes already given the same host state are also given that host. Any remaining nodes with "*" are given the unassigned state.

SI 1.2 Maximum-parsimony reconstruction with within-host diversity penalty

A naive, rigorous, maximum-parsimony reconstruction is straightforward; the Sankoff algorithm (Sankoff, 1975) provides a general method. However, there are two limitations to such an approach. Firstly, it cannot handle the unassigned state that we propose. Secondly, in attempting to minimise the number of state changes (which, in our case, correspond to infection events) sinmple parsimony will sometimes make an unrealistic reconstruction of a single introduction to a host in cases where so much diversity exists within the sample taken from that host that two or more separate introductions is much more plausible.

We deal with the first limitation by treating "unassigned" as a separate state, which is given to any reference sequences and blacklisted tips. We also assume that the MRCA lineage of the entire phylogeny was not present in any host in the study population and hence also has the unassigned state. This can always be achieved by the selection of a suitable outgroup. The down phase of the Sankoff algorithm then skips the determination of the root node state by parsimony and conditions the reconstruction on that node having the unassigned state. This is because transitions happening above the root node are not counted when costing a tree, and hence the algorithm can reduce the total cost by placing the root within a host in the study population, which will be unrealistic in many datasets. We are interested only in minimising the number of infection events involving members of the study population as recipients; we do not attempt to quantify the number of infections of unsampled individuals with this procedure. As a result, transitions to the unassigned state have no cost.

The issue of unrealistic amounts of within-host diversity is dealt with by applying an additional penalty to the parsimony cost of an infection event, which increases with the amount of within-host diversity occurring in the branches descended from each node. This makes the parsimony reconstruction edge-dependent, meaning that transitions have different costs at different locations on the tree, but the Sankoff algorithm is still applicable in these circumstances (Erdős & Székely,

1994). In particular, if n is a node and h a host, suppose $l(h,n)$ is the sum of the branch lengths of the subtree obtained by pruning the subtree rooted at n of all tips from hosts other than h (or infinity if there are not such tips). Then we set the cost $c(h,n)$ of transitioning to h along the branch ending in n (from any other host, or the unassigned state) to:

$$c(n,h) = 1 + k \times l(n,h)$$

where k is a tunable constant. Thus if $l(h,n)$ is large, it may be less expensive to reconstruct two infection events to h, further down the tree, rather than a single one at n (see figure S1). For two clades from the same host, k can be interpreted as the the reciprocal of the minimum patristic distance between the two clade MRCA nodes that would suggest that each was the result of a separate infection event. Setting k to zero recovers standard parsimony with the unassigned state included, and can be safely done if it is known that no superinfection events are present. (It is not recommended that k be used to try to separate lineages in a patient that are the results of different infection events from the same source. Parsimony should naturally do this if that source is sampled, and if they are not then the *phyloscanner* approach lacks the resolution to reliably do this on a large scale.) As a general rule of thumb, a good value of k is the reciprocal of a patristic distance so large that it would be surprising to encounter a host with an infection so diverse that a) the infection had a single source and b) a phylogeny built from pathogen sequences from just that host had a branch that long or longer.

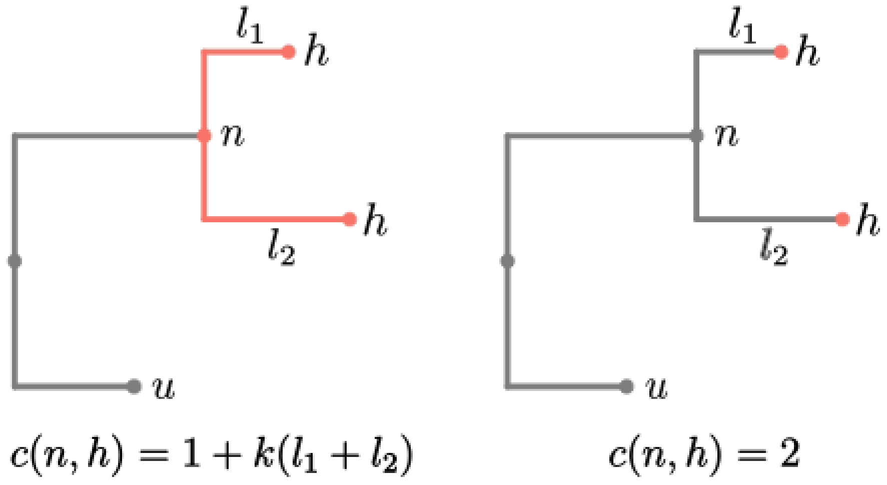

Figure S1 - Parsimony costs for two reconstructions of host states onto the same tree. On the left a single infection event for the host h (from the unassigned state u) is reconstructed, while on the right two separate events are. The penalty for the single introduction is k multiplied by the sum of the branch lengths of the subtree rooted at the node n, i.e. $l_1 + l_2$. As a result, the dual infection scenario is preferred when $k(l_1 + l_2) > 1$.

The parsimony costs given here can, in some circumstances, result in multiple reconstructions of the whole tree having the same cost. For example, in figure S1, the top row will always have the same cost (because the transition to the unassigned state has no cost, it is equally parsimonious to transition from the green host to it along the branch leading to the starred node, or to stay in that green state). In addition, if $k = 0$ then the bottom row also have the same cost. (This is not true if k is greater than zero because those reconstructions will be penalised for greater within-host diversity.) This situation generally arises at nodes whose children and parent all have different most parsimonious states. The normal behaviour of *phyloscanner* is to make the reconstruction in the top left; the starred node is reconstruction as unassigned. The reason for this is that it allows the adjacency relationship (see section S1.4) to apply to all possible pairs of hosts amongst the

neighbours of that node, so all (all three, in figure S2) are inferred to have a transmission relationship with each other. In a relationship diagram, they would appear as the triangle in figure 7 of the main text. It is also the most parsimonious reconstruction that assigns the smallest number of nodes to each host. Such areas of the phylogeny should be treated as similar to branches connecting nodes in different host subgraphs: they may, or may not, involve an unsampled intermediate host and the probability of this will increase as the branch lengths involved do. We make this decision because, when several transmission histories are equally parsimonious, we feel the the consequences of making a random choice are more serious than those of leaving the situation ambiguous. Future refinements to the reconstruction procedure, using parsimony or other methods, may be able to more firmly resolve topological arrangements of this sort.

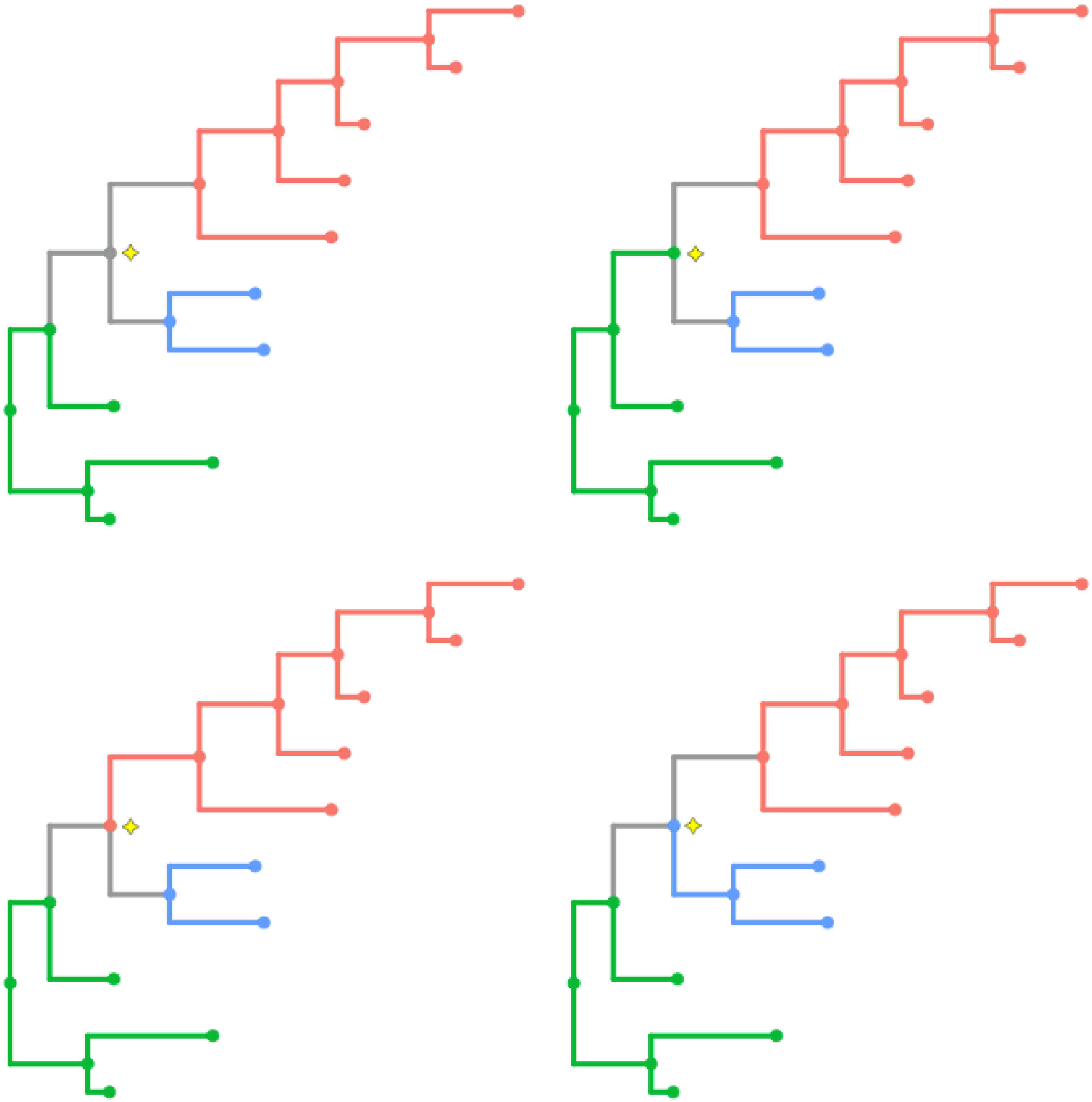

Figure S2 - Parsimony reconstructions with equal costs. The reconstruction of the starred node to the unassigned state (top left) and to the green host (top right) are equally parsimonious. If $k = 0$, but not otherwise, its reconstruction to the red or blue hosts (bottom row) also have the same cost. The normal behaviour of `phyloscanner` is to make the "unassigned" reconstruction in the top left.

SI 1.3 Parsimony reconstructions for the identification of contaminant reads

The Sankoff parsimony reconstruction penalises the reconstruction of a single infection of any individual based on the amount of within-host diversity that such a reconstruction would involve. If such a penalty is sufficiently large, two or more infection events are reconstructed instead. This allows the detection of not only genuine dual infections, but contaminant reads as well: if a small number of reads are very distant in the phylogeny to the bulk of the diversity in a single individual, then contamination is a likely explanation, and the algorithm will identify such reads. To make use of this, `phyloscanner` allows for identification of likely contaminants by, for each individual, pruning the full phylogeny until only tips from that individual and an outgroup remain, and then performing the parsimony reconstruction with a value of k chosen as it would be in detecting multiple infections. In this reconstruction the only valid states are the state for the individual in question and the unassigned state. If this results in the reconstruction of multiple infections for the individual, the read counts for the tips making up each of these "infections" are examined and tips belonging to those infections that fail to meet a specified numerical threshold are reported. These tip labels can then be removed from the analysis if the tree is rebuilt, or blacklisted so that they are not considered in a full parsimony reconstruction using the full set of individuals even if the same tree is kept. If multiple infections remain after this process for removing contaminants, this constitutes evidence that the individual is genuinely multiply infected (in that window of the genome).

SI 1.4 The collapsed tree

The "collapsed tree" is obtained from the annotated phylogeny by collapsing all sets of nodes with the same annotation which form connected regions of the tree, including those with the unassigned annotation, to single nodes (see Fig. S3). The collapsed tree is, if the phylogeny and reconstruction is correct, a partial transmission tree (partial due to the existence of unassigned areas) which treats separate introductions to the same host as separate nodes.

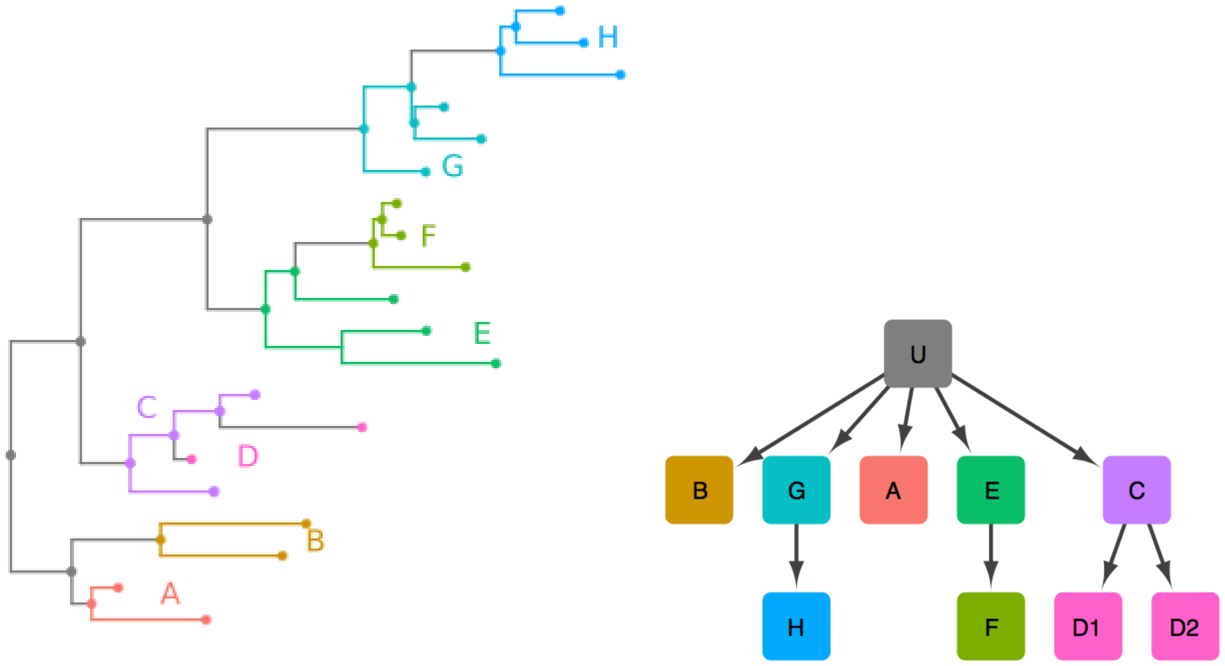

Figure S3 - How a phylogeny with hosts reconstructed on internal nodes can be "collapsed" to a visualisation of the transmission process. Left: A phylogeny, with node colours

representing the hosts A-H which each lineage infected. Tip hosts are known from the data, while internal node hosts are determined by ancestral state reconstruction. Nodes with the grey colour are reconstructed to the “unassigned” state. Coloured branches connect nodes with the same hosts and indicate membership of the same subgraph. Grey branches connect subgraphs and “unassigned” nodes to each other. Right: the visualisation of the transmission process (“collapsed tree”) obtained from this coloured phylogeny. Each subgraph forms a node in this tree (arrow lengths are not meaningful). Where there is more than one subgraph for a single individual (such as for D here), multiple nodes appear in the collapsed tree, representing an infection by multiple lineages.

As `phyloscanner` is usually used to reconstruct internal node states for multiple trees, either from different genome windows or from bootstrap or posterior replicates, there may be many collapsed trees in the output. Ideally, these could be summarised in a single diagram, but no procedure to produce one currently exists. Difficulties in producing one revolve around, firstly, the existence of varying number of collapsed tree nodes from one host in different phylogenies, secondly, the existence of unassigned regions, and thirdly, for data consisting of short reads from genome windows, the potential complete absence of some hosts from some windows due to uneven sequencing.

In the absence of a summary tree method, we concentrate on identifying the variation in the relationship between each pair of hosts across the different phylogenies. We propose four ways in which a pair can be related:

- *Distance*, defined as the minimum distance between a collapsed tree node from one of the pair and a node from the other.
- *Adjacency,* whether any pair of nodes from the two individuals in the collapsed tree are either directly connected to each other or connected through unassigned nodes only. This is the default way in which we establish that a topological relationship between hosts exists.
- *Contiguity*, whether all nodes from the two individuals form a connected region of the collapsed tree, possibly with some unassigned nodes. This is an alternative, more stringent means of identifying a topological relationship.
- *Topological classification*, how nodes from the pair are arranged in the collapsed tree in relation to each other.

The four categories of topological classification are:

- *Single ancestry*, in which there is only collapsed tree node from one host and that host is a descendant of a node from the other.
- *Multiple ancestry*, in which there are multiple tree nodes from one host but all are descendants of a node or nodes from the other.
- *No ancestry*, in which no node from either individual is an ancestor of a node from the other.
- *Complex cases*, where none of the above are true.

See Figure S4 for an illustration of these.

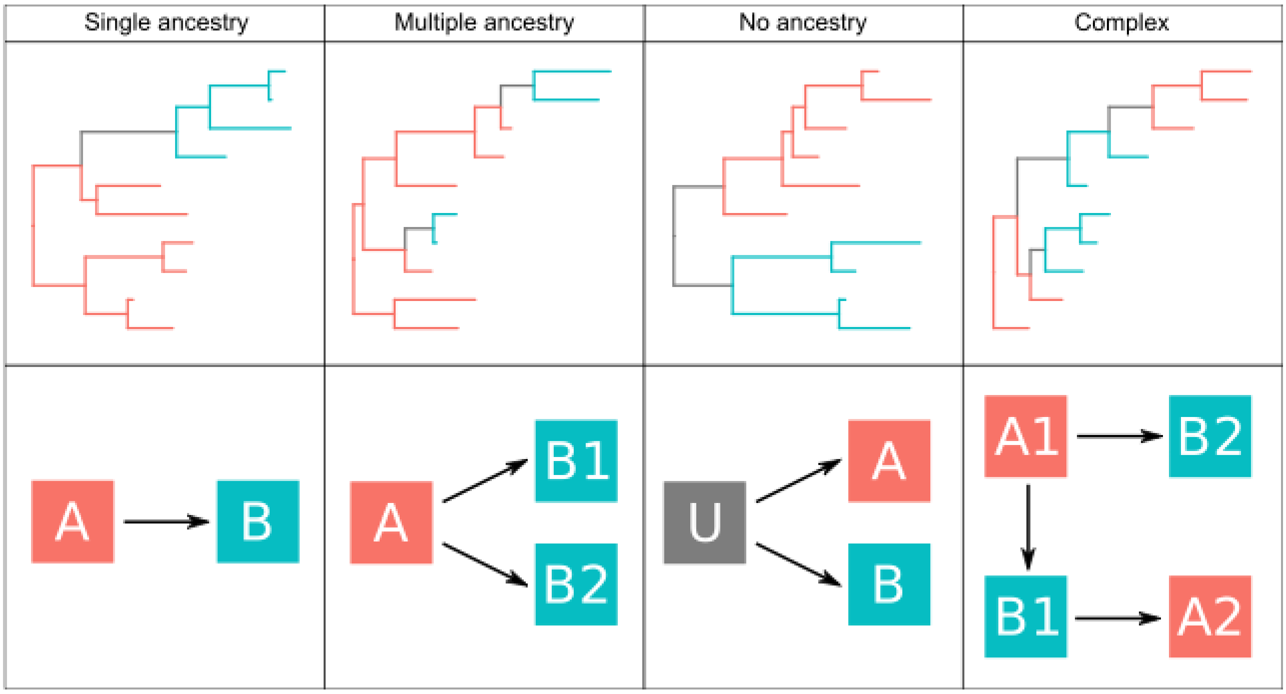

Figure S4 - Examples of the four classes of topological relationship between two infected individuals. The phylogeny with hosts reconstructed to nodes is above, and the collapsed trees below.

We can summarise relationships across all the trees in our sample. For the results presented in this paper, we inferred links between hosts on a single tree if they are both adjacent and within a distance threshold of each other. Each of those links can be classified into six categories (the four above, with the two ancestry categories appearing in both directions). Because the "multiple ancestry" relationship is weaker evidence of the direction of transmission than "single ancestry", by default `phyloscanner` will merge it with "complex" relationship and not infer a direction of transmission for trees in which it occurs, but this behaviour can be changed by the user. We then have, over all trees, a count of how often each pair of hosts are linked, and what the topology suggests about the relationship between them in each case.

SI 2: HIV-1 Data Sequenced with the Roche 454 Platform: Generation and `phyloscanner` Analysis

Following RNA extraction, four BEEHIVE study samples were amplified and sequenced according to the protocol of Gall *et al.* Briefly, amplicons were pooled in equimolar amounts. Single-stranded DNA libraries were prepared from 500 ng DNA with the GS FLX Titanium Rapid Library Preparation Kit according to the manufacturer's instructions, using one of the 48 Multiplex Identifier (MID) adaptors for each sample. Sequencing was performed using the Genome Sequencer FLX Instrument and GS FLX Titanium series reagents.

`phyloscanner` takes as input mapped reads. For any mapping of reads, to maximise accuracy it is desirable to first construct a reference as close as possible to the expected consensus of the reads. Given that the relatively high error rate of the Roche 454 platform complicates both *de novo* assembly and the calling of a preliminary consensus from preliminary mapping, and that Illumina sequence data was also available for the same patients, for simplicity we constructed each patient's reference by applying `IVA` and `shiver` to the Illumina data as described in Methods. The Roche 454 reads were mapped to the reference using `BWA`.

phyloscanner was run on the mapped reads for these four samples, using 54 windows each of length 320 bp (defined with respect to coordinates of HXB2) and each overlapping with its neighbour by 160 bp, skipping the window wholly overlapping variable loops 1 and 2, exactly as for the BEEHIVE Illumina data. In Figure S5 we show the resulting phylogenies for three illustrative genomic windows.

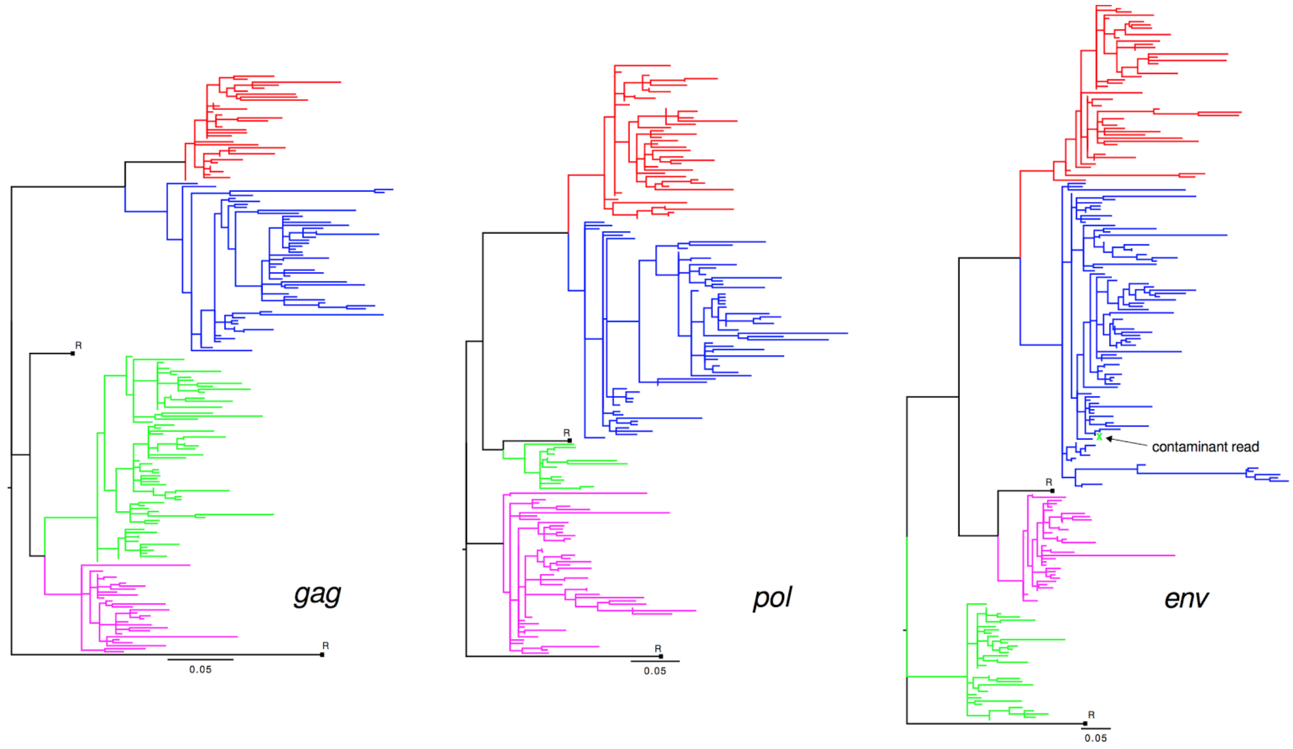

Figure S5 - phyloscanner output phylogenies for Roche 454 sequence data from four HIV-1 patients, for windows in the *gag* (left), *pol* (middle) and *env* (right) genes. Tips are coloured by patient, as are all nodes assigned to that patient by ancestral reconstruction, and the branches connecting these tips and nodes; a solid block of colour therefore defines a single subgraph for one patient (see main text). The patients each have a single subgraph, indicating single infections (i.e. no multiple infections). No subgraph from one patient is descended from or ancestral to a subgraph from another patient, which is evidence against one of these patients infecting another.

SI 3: Measuring Recombination

phyloscanner calculates a basic metric of recombination that aims to detect a single crossover point. The metric is calculated, for each sample's reads in each window, as follows. For each combination of three reads, with one the putative recombinant and the other two the parents, and each possible crossover point, d_L is defined to be the (signed) difference in Hamming distance between the recombinant and parent 1, and the recombinant and parent 2, to the left of the crossover point. d_R is defined similarly to the right of the crossover point. We maximise the difference between d_L and d_R (over all possible sets of three sequences and all possible crossover points), take the smaller of the two absolute values, and normalise it by half the length of the alignment of sequences. The resulting metric is constrained to be between 0 and 1, inclusive. The maximum possible score of 1 is obtained if and only if the two parents disagree at every site, the crossover point is exactly in the middle, and either side of the crossover point the recombinant agrees perfectly with one of the parents, for example

```
AAAAAAA
AAAACCC
CCCCCCC
```

If the above pattern is observed only at polymorphic sites, with a fraction x of sites in the window being polymorphic (and 1-x being conserved), the score will be x. Figure S6 shows the three reads giving rise to the highest value of the metric in the dataset presented in the results section *HIV-1 sequenced with Illumina Miseq*.

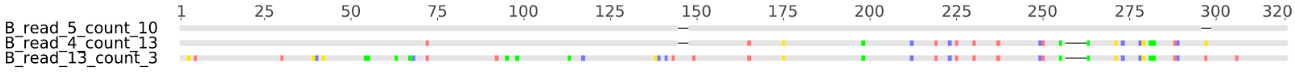

Figure S6 - The HIV-1 reads displaying the greatest amount of recombination. In the sequence names, the count indicates the number of times that specific sequence was found amongst the mapped reads; the sequences are then ranked by count, so that read 1 is the most common. These three reads from the dually infected individual B give rise to the maximum value of the recombination metric in this dataset: 0.125. Bases are grey where they agree with the first sequence, and coloured otherwise. Thin horizontal black lines inside a sequence indicate deletions.

This simple metric looks only for single crossover points, and is agnostic about the biology of recombination, considering only Hamming distances. Included in `phyloscanner` output are the reads from each chosen window, after extraction, processing and alignment; these can therefore be used as the starting point for more sophisticated investigations of recombination (which is an area of research in its own right).

SI 4: Members of the BEEHIVE Cohorts

Swiss HIV cohort

The member of the Swiss HIV Cohort are: Aubert V, Battegay M, Bernasconi E, Böni J, Braun DL, Bucher HC, Burton-Jeangros C, Calmy A, Cavassini M, Dollenmaier G, Egger M, Elzi L, Fehr J, Fellay J, Furrer H (Chairman of the Clinical and Laboratory Committee), Fux CA, Gorgievski M, Günthard H (President of the SHCS), Haerry D (deputy of “Positive Council”), Hasse B, Hirsch HH, Hoffmann M, Hösli I, Kahlert C, Kaiser L, Keiser O, Klimkait T, Kouyos R, Kovari H, Ledergerber B, Martinetti G, Martinez de Tejada B, Marzolini C, Metzner K, Müller N, Nadal D, Nicca D, Pantaleo G, Rauch A (Chairman of the Scientific Board), Regenass S, Rudin C (Chairman of the Mother & Child Substudy), Schöni-Affolter F (Head of Data Centre), Schmid P, Speck R, Stöckle M, Tarr P, Trkola A, Vernazza P, Weber R, Yerly S.

ATHENA cohort (The Netherlands)

CLINICAL CENTRES:
* denotes site coordinating physician
Academic Medical Centre of the University of Amsterdam (AMC-UvA): *HIV treating physicians:* M. van der Valk*, S.E. Geerlings, M.H. Godfried, A. Goorhuis, J.W. Hovius, J.T.M. van der Meer, T.W. Kuijpers, F.J.B. Nellen, DT. van der Poll, J.M. Prins, P. Reiss, H.J. M. van Vugt, W.J. Wiersinga, F.W.M.N. Wit. *HIV nurse consultants:* M. van Duinen, J. van Eden, A.M.H. van Hes, M. Mutschelknauss, H.E. Nobel, F.J.J. Pijnappel, A.M. Weijsenfeld. *HIV clinical virologists/chemists:* S. Jurriaans, N.K.T. Back, H.L. Zaaijer, B. Berkhout, M.T.E. Cornelissen, C.J.

Schinkel, K.C. Wolthers. **Admiraal De Ruyter Ziekenhuis, Goes:** *HIV treating physicians:* M. van den Berge, A. Stegeman. *HIV nurse consultants:* S. Baas, L. Hage de Looff. *HIV clinical virologists/chemists:* B Wintermans, J Veenemans. **Catharina Ziekenhuis, Eindhoven:** *HIV treating physicians:* M.J.H. Pronk*, H.S.M. Ammerlaan. *HIV nurse consultants:* E.S. de Munnik, H.A.M. van Beek. *HIV clinical virologists/chemists:* A.R. Jansz, J. Tjhie, M.C.A. Wegdam, B. Deiman, V. Scharnhorst. **Elisabeth-TweeSteden Ziekenhuis, Tilburg:** *HIV treating physicians:* M.E.E. van Kasteren*, A.E. Brouwer. *HIV nurse consultants:* R. van Erve, B.A.F.M. de Kruijf-van de Wiel, S.Keelan-Pfaf, B. van der Ven. *Data collection:* B.A.F.M. de Kruijf-van de Wiel, B. van der Ven. *HIV clinical virologists/chemists:* A.G.M. Buiting, P.J. Kabel, D.Versteeg. **Erasmus MC, Rotterdam:** *HIV treating physicians:* M.E. van der Ende*, H.I. Bax, E.C.M. van Gorp, J.L. Nouwen, B.J.A. Rijnders, C.A.M. Schurink, A. Verbon, T.E.M.S. de Vries-Sluijs, N.C. de Jong-Peltenburg. *HIV nurse consultants:* N. Bassant, J.E.A. van Beek, M. Vriesde, L.M. van Zonneveld. *Data collection:* H.J. van den Berg-Cameron, J. de Groot, M. de Zeeuw-de Man. *HIV clinical virologists/chemists:* C.A.B. Boucher, M.P.G Koopmans, J.J.A van Kampen, S.D. Pas. **Flevoziekenhuis, Almere:** *HIV treating physicians:* J. Branger*, A. Rijkeboer-Mes. *HIV nurse consultant:* C.J.H.M. Duijf-van de Ven. **HagaZiekenhuis, Den Haag:** *HIV treating physicians:* E.F. Schippers*, C. van Nieuwkoop. *HIV nurse consultants:* J.M. van IJperen, J. Geilings. *Data collection:* G. van der Hut. *HIV clinical virologist/chemist:* N.D. van Burgel. **Hiv Focus Centrum (DC Klinieken):** *HIV treating physicians:* A. van Eeden*. *HIV nurse consultants:* W. Brokking, M. Groot, L.J.M. Elsenburg. *HIV clinical virologists/chemists:* M. Damen, I.S. Kwa. **HMC (Haaglanden Medisch Centrum), Den Haag:** *HIV treating physicians:* E.M.S. Leyten*, L.B.S. Gelinck. *HIV nurse consultants:* A.Y. van Hartingsveld, C. Meerkerk, G.S. Wildenbeest. *HIV clinical virologists/chemists:* E. Heikens. **Isala, Zwolle:** *HIV treating physicians:* P.H.P. Groeneveld*, J.W. Bouwhuis, A.J.J. Lammers. *HIV nurse consultants:* S. Kraan, A.G.W. van Hulzen. *Data collection:* G.L. van der Bliek, P.C.J. Bor. *HIV clinical virologists/chemists:* P. Bloembergen, M.J.H.M. Wolfhagen, G.J.H.M. Ruijs. **Leids Universitair Medisch Centrum, Leiden:** *HIV treating physicians:* F.P. Kroon*, M.G.J. de Boer, H. Scheper, H. Jolink, A.M. Vollaard. *HIV nurse consultants:* W. Dorama, N. van Holten. *HIV clinical virologists/chemists:* E.C.J. Claas, E. Wessels. **Maasstad Ziekenhuis, Rotterdam:** *HIV treating physicians:* J.G. den Hollander*, K. Pogany, A. Roukens. *HIV nurse consultants:* M. Kastelijns, J.V. Smit, E. Smit, D. Struik-Kalkman, C. Tearno. *Data collection:* T. van Niekerk. *HIV clinical virologists/chemists:* O. Pontesilli. **Maastricht UMC+, Maastricht:** *HIV treating physicians:* S.H. Lowe*, A.M.L. Oude Lashof, D. Posthouwer. *HIV nurse consultants:* R.P. Ackens, K. Burgers, J. Schippers. *Data collection:* B. Weijenberg-Maes. *HIV clinical virologists/chemists:* I.H.M. van Loo, T.R.A. Havenith. **MC Slotervaart, Amsterdam:** *HIV treating physicians:* J.W. Mulder*, S.M.E. Vrouenraets, F.N. Lauw. *HIV nurse consultants:* M.C. van Broekhuizen, D.J. Vlasblom. *HIV clinical virologists/chemists:* P.H.M. Smits. **MC Zuiderzee, Lelystad:** *HIV treating physicians:* S. Weijer*, R. El Moussaoui. *HIV nurse consultant:* A.S. Bosma. **Medisch Centrum Leeuwarden, Leeuwarden:** *HIV treating physicians:* M.G.A.van Vonderen*, D.P.F. van Houte, L.M. Kampschreur. *HIV nurse consultants:* K. Dijkstra, S. Faber. *HIV clinical virologists/chemists:* J Weel. **Medisch Spectrum Twente, Enschede:** *HIV treating physicians:* G.J. Kootstra*, C.E. Delsing. *HIV nurse consultants:* M. van der Burg-van de Plas, H. Heins. *Data collection:* E. Lucas. **Noordwest Ziekenhuisgroep, Alkmaar:** *HIV treating physicians:* W. Kortmann*, G. van Twillert*, R. Renckens. *HIV nurse consultant and data collection:* D. Ruiter-Pronk, F.A. van Truijen-Oud. *HIV clinical virologists/chemists:* J.W.T. Cohen Stuart, E.P. IJzerman, R. Jansen, W. Rozemeijer W. A. van der Reijden. **OLVG, Amsterdam:** *HIV treating physicians:* K. Brinkman*, G.E.L. van den Berk, W.L. Blok, P.H.J. Frissen, K.D. Lettinga W.E.M. Schouten, J. Veenstra. *HIV nurse consultants:* C.J. Brouwer, G.F. Geerders, K. Hoeksema, M.J. Kleene, I.B. van der Meché, M. Spelbrink, A.J.M. Toonen, S. Wijnands. *HIV clinical virologists:* D. Kwa. *Data collection:* R. Regez (coordinator). **Radboudumc, Nijmegen:** *HIV treating physicians:* R. van Crevel*, M. Keuter, A.J.A.M. van der Ven, H.J.M. ter Hofstede, A.S.M. Dofferhoff, J. Hoogerwerf. *HIV nurse consultants:* K.J.T. Grintjes-Huisman, M. de Haan, M. Marneef, A. Hairwassers. *HIV clinical virologists/chemists:* J. Rahamat-Langendoen, F.F. Stelma. *HIV clinical*

pharmacology consultant: D. Burger. **Rijnstate, Arnhem:** *HIV treating physicians:* E.H. Gisolf*, R.J. Hassing, M. Claassen. *HIV nurse consultants:* G. ter Beest, P.H.M. van Bentum, N. Langebeek. *HIV clinical virologists/chemists:* R. Tiemessen, C.M.A. Swanink. **Spaarne Gasthuis, Haarlem:** *HIV treating physicians:* S.F.L. van Lelyveld*, R. Soetekouw. *HIV nurse consultants:* L.M.M. van der Prijt, J. van der Swaluw. *Data collection:* N. Bermon. *HIV clinical virologists/chemists:* W.A. van der Reijden, R. Jansen, B.L. Herpers, D.Veenendaal. **Medisch Centrum Jan van Goyen, Amsterdam:** *HIV treating physicians:* D.W.M. Verhagen. *HIV nurse consultants:* M. van Wijk. **Universitair Medisch Centrum Groningen, Groningen:** *HIV treating physicians:* W.F.W. Bierman*, M. Bakker, J. Kleinnijenhuis, E. Kloeze, Y. Stienstra, K.R. Wilting, M. Wouthuyzen-Bakker. *HIV nurse consultants:* A. Boonstra, P.A. van der Meulen, D.A. de Weerd. *HIV clinical virologists/chemists:* H.G.M. Niesters, C.C. van Leer-Buter, M. Knoester. **Universitair Medisch Centrum Utrecht, Utrecht:** *HIV treating physicians:* A.I.M. Hoepelman*, J.E. Arends, R.E. Barth, A.H.W. Bruns, P.M. Ellerbroek, T. Mudrikova, J.J. Oosterheert, E.M. Schadd, M.W.M. Wassenberg, M.A.D. van Zoelen. *HIV nurse consultants:* K. Aarsman, D.H.M. van Elst-Laurijssen, I. de Kroon, C.S.A.M. van Rooijen. *Data collection:* M. van Berkel, C.S.A.M. van Rooijen. *HIV clinical virologists/chemists:* R. Schuurman, F. Verduyn-Lunel, A.M.J. Wensing. **VUmc, Amsterdam:** *HIV treating physicians:* E.J.G. Peters*, M.A. van Agtmael, M. Bomers. *HIV nurse consultants:* M. Heitmuller, L.M. Laan. *HIV clinical virologists/chemists:* C.W. Ang, R. van Houdt, A.M. Pettersson, C.M.J.E. Vandenbroucke-Grauls.
COORDINATING CENTRE:
Director: P. Reiss. *Data analysis:* D.O. Bezemer, A.I. van Sighem, C. Smit, F.W.M.N. Wit, T.S. Boender. *Data management and quality control:* S. Zaheri, M. Hillebregt, A. de Jong. *Data monitoring:* D. Bergsma, S. Grivell, A. Jansen, M. Raethke, R. Meijering, T. Rutkens. *Data collection:* L. de Groot, M. van den Akker, Y. Bakker, M. Bezemer, E. Claessen, A. El Berkaoui, J. Geerlinks, J. Koops, E. Kruijne, C. Lodewijk, R. van der Meer, L. Munjishvili, F. Paling, B. Peeck, C. Ree, R. Regtop, Y. Ruijs, M. Schoorl, A. Timmerman, E. Tuijn, L. Veenenberg, S. van der Vliet, A. Wisse, E.C. de Witte, T. Woudstra. *Patient registration:* B. Tuk.

Antwerp cohort (Belgium)

Data extraction for the Antwerp Cohort is done by Maartje Van Frankenhuijsen, MD.

PRIMO cohort (France):

Région Sud–Est:

- Thierry ALLEGRE, Centre hospitalier général d'Aix en Provence, Service d'Hématologie
- Djamila MAKHLOUFI, Jean-Michel LIVROZET, Pierre CHIARELLO, Mathieu GODINOT, Florence BRUNEL-DALMAS, Sylvie GIBERT, Hôpital Edouard Herriot de Lyon, Immunologie Clinique
- Christian TREPO, Dominique PEYRAMOND, Patrick MIAILHES, Joseph KOFFI, Valérie THOIRAIN, Corinne BROCHIER, Thomas BAUDRY, Sylvie PAILHES, Lyon La Croix Rousse, Services d'Hépato-Gastroentérologie et des Maladies Infectieuses
- Alain LAFEUILLADE, Gisèle PHILIP, Gilles HITTINGER, Assi ASSI, Véronique LAMBRY, Hôpital Font-Pré de Toulon, Médecine Interne, Hémato-Infectiologie
- Eric ROSENTHAL, Alissa NAQVI, Brigitte DUNAIS, Eric CUA, Christian PRADIER, Jacques DURANT, Aline JOULIE, Hôpital L'Archet, Nice, Service de Médecine Interne, Maladies Infectieuses et Tropicales
- Denis QUINSAT, Serge TEMPESTA, Centre Hospitalier d'Antibes, Service de Médecine Interne
- Isabelle RAVAUX, Hôpital de la Conception de Marseille, Service des Maladies Infectieuses
- Isabelle POIZOT MARTIN, Olivia FAUCHER, Nicolas CLOAREC, Hôpital Sainte Marguerite de Marseille, Unité d'Hématologie

- Hélène CHAMPAGNE, Centre Hospitalier de Valence, Maladies Infectieuses et Tropicales
- Gilles PICHANCOURT, Centre Hospitalier Henri Duffaut d'Avignon, Service Hématologie Maladies Infectieuses

<u>Région Sud-Ouest:</u>

- Philippe MORLAT, Thierry PISTONE, Fabrice BONNET, Patrick MERCIE, Isabelle FAURE, Mojgan HESSAMFAR, Denis MALVY, Denis LACOSTE, Marie-Carmen PERTUSA, Marie-Anne VANDENHENDE, Noëlle BERNARD, François PACCALIN, Cédric MARTELL, Julien ROGER-SCHMELZ, Marie-Catherine RECEVEUR, Pierre DUFFAU, Denis DONDIA, Emmanuel RIBEIRO, Sabrina CALTADO, Hôpital Saint André de Bordeaux, Médecine Interne
- Didier NEAU, Michel DUPONT; Hervé DUTRONC, Frédéric DAUCHY, Charles CAZANAVE, Thierry PISTONE, Marc-Olivier VAREIL, Thierry PISTONE, Gaétane WIRTH, Séverine LE PUIL, Hôpital Pellegrin de Bordeaux, Maladies Infectieuses.
- Jean-Luc PELLEGRIN, Isabelle RAYMOND, Jean-François VIALLARD, Severin CHAIGNE DE LALANDE, Hôpital Haut Lévèque de Bordeaux, Médecine Interne et Maladies Infectieuses
- Daniel GARIPUY, Hôpital Joseph Ducuing de Toulouse, Médecine Interne
- Pierre DELOBEL, Martine OBADIA, Lise CUZIN, Muriel ALVAREZ, Noemie BIEZUNSKI, Lydie PORTE, Patrice MASSIP, Alexa DEBARD, Florence BALSARIN, Myriam LAGARRIGUE, Hôpital Purpan de Toulouse, SMIT-CISIH
- François PREVOTEAU DU CLARY, Christian AQUILINA, Cité de la santé Toulouse
- Jacques REYNES, Vincent BAILLAT, Corinne MERLE, Vincent LEMOING, Nadine ATOUI, Alain MAKINSON, Jean Marc JACQUET, Christina PSOMAS, Christine TRAMONI, Hôpital Gui de Chauliac de Montpellier, Service des Maladies Infectieuses et Tropicales
- Hugues AUMAITRE, Mathieu SAADA, Marie MEDUS, Martine MALET, Aurélia EDEN, Ségolène NEUVILLE, Milagros FERREYRA, Martine MALET, Hôpital Saint Jean de Perpignan, Service des Maladies Infectieuses
- Albert SOTTO, Claudine BARBUAT, Isabelle ROUANET, Didier LEUREILLARD, Jean-Marc MAUBOUSSIN, Catherine LECHICHE, Régine DONSESCO, CHU de Nîmes-Caremeau, Service des Maladies Infectieuses et Tropicales.

<u>Antilles:</u>

-André CABIE, Sylvie ABEL, Sandrine PIERRE-FRANCOIS, Anne-Sophie BATALA, Christophe CERLAND, Camille RANGOM, Nadine THERESINE, CHU Fort de France, Hôpital de Jour
-Bruno HOEN, Isabelle LAMAURY, Isabelle FABRE, Kinda SCHEPERS, Elodie CURLIER, Rachida OUISSA, CHU de Pointe à Pitre/ABYMES, Service de Dermatologie / Maladies Infectieuses
-Catherine GAUD, Carole RICAUD, Roland RODET, Guillaume WARTEL, Carmele SAUTRON, CHU de la Reunion, site Felix Guyon, Service d'Immunologie

<u>Région Est:</u>

- Geneviève BECK-WIRTH, Catherine MICHEL, Charles BECK, Jean-Michel HALNA, Jakub KOWALCZYK, Meryem BENOMAR, Hôpital Emile Muller de Mulhouse, Hématologie Clinique
- Christine DROBACHEFF-THIEBAUT, Catherine CHIROUZE, Jean-François FAUCHER, François PARCELIER, Adeline FOLTZER, Cécile HAFFNER-MAUVAIS, Mathieu HUSTACHE MATHIEU, Aurélie PROUST - Hôpital St Jacques de Besançon, Service des Maladies Infectieuses et de Dermatologie
- Lionel PIROTH, Pascal CHAVANET, Michel DUONG, Marielle BUISSON, Anne WALDNER, Sophie MAHY, Sandrine GOHIER, Delphine CROISIER, Hôpital du Bocage de Dijon, Service des Maladies Infectieuses
- Thierry MAY, Mikael DELESTAN, Marie ANDRE, CHU de Vandoeuvre-lès-Nancy, Hôpital de Brabois, Service des Maladies Infectieuses et Tropicales
- Mahsa MOHSENI ZADEH, Martin MARTINOT, Béatrice ROSOLEN, Anne PACHART, Hôpital Louis PASTEUR de Colmar, Service d'Immunologie Clinique
- Benoît MARTHA, Noëlle JEUNET, Centre Hospitalier William Morey de Chalon Sur Saône, Service de Médecine Interne

- David REY, Christine CHENEAU, Maria PARTISANI, Michèle PRIESTER, Claudine BERNARD-HENRY, Maria PARTISANI, Marie-Laure BATARD, Patricia FISCHER, Service le Trait d'Union, Hôpitaux Universitaires de Strasbourg
- Jean-Luc BERGER, Isabelle KMIEC, Hôpital Robert Debré, Service des Maladies Infectieuses, Reims.

Région Nord:

- Olivier ROBINEAU, Thomas HULEUX, Faïza AJANA, Isabelle ALCARAZ, Christophe ALLIENNE, Véronique BACLET, Agnès MEYBECK, Michel VALETTE, Nathalie VIGET, Christophe ALLIENNE, Emmanuelle AISSI, Raphael BIEKRE, Pauline CORNAVIN, Centre Hospitalier DRON de Tourcoing, Service de Maladies Infectieuses
- Dominique MERRIEN, Jean-Christophe SEGHEZZI, Moise MACHADO, Centre Hospitalier de Compiègne, Service de Médecine Interne
- Georges DIAB, C H de la Haute Vallée de l'Oise de Noyon, Service de Médecine

Région Ouest:

- François RAFFI, Bénédicte BONNET, Clotilde ALLAVENA, Olivier GROSSI, Véronique RELIQUET, Eric BILLAUD, Cecile BRUNET, Sabelline BOUCHEZ, Pascale MORINEAU-LE HOUSSINE, Fabienne SAUSER, David BOUTOILLE, Michel BESNIER, Hervé HUE, Nolwenn Hall, Delphine BROSSEAU, Hôtel-Dieu de Nantes, CISIH Médecine Interne
- Faouzi SOUALA, Christian MICHELET, Pierre TATTEVIN, Cédric ARVIEUX, Matthieu REVEST, Helene LEROY, Jean-Marc CHAPPLAIN, Matthieu DUPONT, Fabien FILY, Solène PATRA-DELO, Céline LEFEUVRE, CHRU Pontchaillou de Rennes, Clinique des Maladies Infectieuses
- Louis BERNARD, Frédéric BASTIDES, Pascale NAU, Hôpital Bretonneau de Tours, Service des maladies Infectieuses
- Renaud VERDON, Arnaud DE LA BLANCHARDIERE, Anne MARTIN, Philippe FERET, CH régional Côte de Nacre de Caen, Service de Maladies Infectieuses
- Loïk GEFFRAY, Hôpital Robert Bisson de Lisieux, Service de Médecine Interne
- Corinne DANIEL, Jennifer ROHAN, Centre Hospitalier La Beauchée de Saint-Brieuc, Médecine Interne et Maladies Infectieuses
- Pascale FIALAIRE, Jean Marie CHENNEBAULT, Valérie Rabier, Pierre ABGUEGUEN, Sami REHAIEM, Centre Hospitalier Régional d'Angers, Service des Maladies Infectieuses
- Odile LUYCX, Mathilde NIAULT, Philippe MOREAU, Centre Hospitalier Bretagne Sud de Lorient, Service d'Hématologie
- Yves POINSIGNON, Marie GOUSSEF, Virginie MOUTON- RIOUX, Centre Hospitalier Bretagne Atlantique de Vannes, Service de Medecine Interne et Maladies Infectieuses
- Dominique HOULBERT, Sandrine ALVAREZ-HUVE, Frédérique BARBE, Sophie HARET, Centre Hospitalier d'Alençon, Médecine 2
- Philippe PERRE,Sophie LEANTEZ-NAINVILLE, Jean-Luc ESNAULT, Thomas GUIMARD, Isabelle SUAUD, Centre Hospitalier Départemental de La Roche sur Yon, Service de Médecine
- Jean-Jacques GIRARD, Véronique SIMONET, Hôpital de Lôches, Service de Médecine Interne
- Yasmine DEBAB, CHU Charles Nicolle de Rouen, Maladies Infectieuses et Tropicales
- Jean-Luc SCHMIT, CHU d'Amiens, Service des Maladies Infectieuses.

Région Centre:

- Christine JACOMET, Hôpital Gabriel-Montpied de Clermont Ferrand, Service des Maladies Infectieuses et Tropicales
- Pierre WEINBERCK, Claire GENET, Pauline PINET, Sophie DUCROIX, Hélène DUROX, Éric DENES, Hôpital DUPUYTREN de Limoges, Maladies Infectieuses et Tropicales
- Bruno ABRAHAM, Centre Hospitalier de Brive, Departement de maladies Infectieuses
- Florence GOURDON, Centre Hospitalier de Vichy, Service de Médecine Interne
- Odile ANTONIOTTI, Centre Hospitalier de Montluçon, Dermatologie

Paris:

- Jean-Michel MOLINA, Samuel FERRET, Caroline LASCOUX-COMBE, Matthieu LAFAURIE, Nathalie COLIN DE VERDIERE, Diane PONSCARME, Nathalie DE CASTRO, Alexandre ASLAN,

Willy ROZENBAUM, Claire PINTADO, François CLAVEL, Olivier TAULERA, Caroline GATEY, Anne-Lise MUNIER, Sandrine GAZAIGNE, Pauline PENOT, Guillaume CONORT, Nathalie LEROLLE, Anne LEPLATOIS, Stéphanie BALAUSINE, Jeannine DELGADO, Hôpital Saint Louis de Paris, Service des Maladies Infectieuses et Tropicales

- Julie TIMSIT, Magda TABET, Hôpital Saint Louis de Paris, Clinique MST
- Laurence GERARD, Hôpital Saint Louis de Paris, Service d'Immunologie Clinique
- Pierre-Marie GIRARD, Odile PICARD, Jürgen TREDUP, Diane BOLLENS, Nadia VALIN, Pauline CAMPA, Julie BOTTERO, Benedicte LEFEBVRE, Muriel TOURNEUR, Laurent FONQUERNIE, Charlotte WEMMERT, Jean-Luc LAGNEAU Hôpital Saint Antoine de Paris , Service des Maladies Infectieuses et Tropicales
- Yazdan YAZDANPANAH, Bao PHUNG, Adriana PINTO, Dorothée VALLOIS, Ornella CABRAS, Françoise LOUNI, G. Hospitalier Bichat-Claude Bernard de Paris, Service de Maladies Infectieuses et Tropicales
- Gilles PIALOUX, Thomas LYAVANC, Valérie BERREBI, Julie CHAS, Sophie LENAGAT, Hopital Tenon de Paris, Service des Maladies Infectieuses
- Agathe RAMI, Myriam DIEMER, Maguy PARRINELLO, Audrey DEPOND, Hôpital Lariboisière de Paris, Service de Médecine Interne A
- Dominique SALMON, Loïc GUILLEVIN, Tassadit TAHI, Linda BELARBI, Pierre LOULERGUE, Olivier ZAK DIT ZBAR, Odile LAUNAY, Benjamin SILBERMANN, Catherine LEPORT, Laura ALAGNA, Marie-Pierre PIETRI, G. H. Cochin de Paris, Département de Médecine Interne
- Anne SIMON, Manuela BONMARCHAND, Naouel AMIRAT, François PICHON, Myriam KIRSTETTER, G. H. Pitié-Salpétrière de Paris, Service de Médecine Interne
- Christine KATLAMA, Marc Antoine VALANTIN, Roland TUBIANA, Fabienne CABY, Luminita SCHNEIDER, Nadine KTORZA, Ruxandra CALIN, Audrey MERLET, Saadia BEN ABDALLAH, G. H. Pitié-Salpétrière de Paris, Service des Maladies Infectieuses
- Laurence WEISS, Martin BUISSON, Dominique BATISSE, Marina KARMOCHINE, Juliette PAVIE, Catherine MINOZZI, Didier JAYLE, Philippe CASTEL, Jean DEROUINEAU, Pascale KOUSIGNAN, Murielle ELIAZEVITCH, Isabelle PIERRE, Lio COLLIAS, Hôpital Européen Georges Pompidou de Paris, Service d'Immunologie Clinique
- Jean-Paul VIARD, Jacques GILQUIN, Alain SOBEL, Laurence SLAMA, Jade GHOSN, Blanka HADACEK, Nugyen THU-HUYN, Audrey MERLET, Lella NAIT-IGHIL, Agnes CROS, Aline MAIGNAN, Hôtel Dieu de Paris, Centre de Diagnostic et Thérapeutique
- Claudine DUVIVIER, Paul Henri CONSIGNY, Fanny LANTERNIER, Michka SHOAI-TEHRANI, Fatima TOUAM, Saadia JERBI, Centre Médical de l'Institut Pasteur de Paris, Service des Maladies Infectieuses
- Loïc BODARD, Corinne JUNG, Institut Mutualiste Montsouris de Paris, Département de Médecine Interne

<u>Région Parisienne:</u>

- Cécile GOUJARD, Yann QUERTAINMONT, Martin DURACINSKY, Olivier SEGERAL, Arnaud BLANC, Delphine PERETTI, Antoine CHERET, Christelle CHANTALAT, Marie Josée DULUCQ, Hôpital de Bicêtre, Médecine Interne
- Yves LEVY, Jean Daniel LELIEVRE, Anne Sophie LASCAUX, Cécile DUMONT, Hôpital Henri Mondor de Créteil, Immunologie Clinique
- François BOUE, Véronique CHAMBRIN, Sophie ABGRALL, Imad KANSAU, Mariem RAHO-MOUSSA, Hôpital Antoine Béclère de Clamart, Médecine Interne et Immunologie Clinique
- Pierre DE TRUCHIS, Aurélien DINH, Benjamin DAVIDO, Dhiba MARIGOT, Huguette BERTHE, Hôpital Raymond Poincaré de Garches, Service des Maladies Infectieuses et Tropicales
- Alain DEVIDAS, Pierre CHEVOJON, Amélie CHABROL, Nouara AGHER, Hôpital de Corbeil-Essonnes, Service Hématologie
- Yvon LEMERCIER, Fabrice CHAIX, Isabelle TURPAULT, Centre Hospitalier Général de Longjumeau, Service de Médecine Interne

- Olivier BOUCHAUD, Patricia HONORE, Hôpital Avicenne de Bobigny, Maladies Infectieuses et Tropicales
- Elisabeth ROUVEIX, Evelyne REIMANN, Hôpital Ambroise Paré de Boulogne, Médecine Interne
- Alix GREDER BELAN, Claire GODIN COLLET, Safia SOUAK, Hôpital du Chesnay, CH Andre Mignot du Chesnay, Maladies Infectieuses et Tropicales
- Emmanuel MORTIER, Martine BLOCH, Anne-Marie SIMONPOLI, Véronique MANCERON, Isabelle CAHITTE, Emmanuel HIRAUX, Erik LAFON, François CORDONNIER ? Ai-feng ZENG, Hôpital Louis Mourier de Colombes, Médecine Interne
- David ZUCMAN, Catherine MAJERHOLC, Dominique BORNAREL, Hôpital Foch de Suresnes , Médecine Interne
- Agnès ULUDAG, Justine GELLEN-DAUTREMER, Agnès LEFORT, Christine BAZIN, Hôpital Beaujon de Clichy, Médecine Interne
- Vincent DANELUZZI, Juliette GERBE, Centre Hospitalier de Nanterre, Service de Médecine Interne
- Vincent JEANTILS, Mélissa COUPARD, Hôpital Jean Verdier de Bondy, Service de Médecine Interne, Unité de Maladies Infectieuses
- Olivier PATEY, Jonas BANTSIMBA, Sophie DELLLION, Pauline CARAUX PAZ, Benoit CAZENAVE, Laurent RICHIER, Centre Hospitalier Intercommunal de Villeneuve St Georges, Médecine Interne
- Valérie GARRAIT, Isabelle DELACROIX, Brigitte ELHARRAR, Laurent RICHIER, Centre Hospitalier Intercommunal de Créteil, Médecine Interne, Hépato-Gastroentérologie
- Daniel VITTECOQ, Claudine BOLLIOT, Hôpital de Bicêtre, Service de Maladies Infectieuses et Tropicales
- Annie LEPRETRE, Hôpital Simone Veil d'Eaubonne, Médecine 2, Consultation ESCALE
- Philippe GENET, Virginie MASSE, Juliette GERBE, Consultation d'Immuno/Hématologie d'Argenteuil
- Véronique PERRONE, Centre Hospitalier François Quesnay de Mantes La Jolie, Service des Maladies Infectieuses
- Jean-Luc BOUSSARD, Patricia CHARDON, Centre Hospitalier Marc Jacquet de Melun, Service de Médecine
- Eric FROGUEL, Philippe SIMON, Sylvie TASSI, Hôpital de Lagny, Service de Médecine Interne.

Scientific Committee:

Véronique AVETTAND FENOEL (Virologie, Necker, Paris), Francis BARIN (Virologie, Tours), Christine BOURGEOIS (INSERM U1184 IMVA, Bicêtre), Fanny CARDON (ANRS), Marie-Laure CHAIX (Virologie, Saint Louis, Paris), Antoine CHERET (Médecine Interne, Paris), Jean François DELFRAISSY (Médecine Interne, Paris), Asma ESSAT (INSERM U1018, Bicêtre), Hugues FISCHER (TRT5), Cécile GOUJARD (Médecine Interne, Bicêtre), Caroline LASCOUX-COMBE (Médecine, Saint Louis, Paris), Camille LECUROUX (INSERM U1184 IMVA, Bicêtre), Laurence MEYER (Santé Publique, INSERM U1018, Bicêtre), Ventzislava PETROV-SANCHEZ (ANRS), Christine ROUZIOUX (Virologie, Necker), Asier SAEZ-CIRION (Institut Pasteur, Paris), Rémonie SENG (Santé Publique, INSERM U1018, Paris).

UK Register of HIV seroconverters:

We would like to thank all the UK Register participants for allowing their routine clinical data to be included. We gratefully acknowledge the work of the members of the Steering Committee and colleagues at the clinical centres. Special thanks go to the following colleagues: Kristin Kuldanek, Scott Mullaney (St Mary's Hospital, London), Carmel Young (Mortimer Market Centre, London), Antonella Zucchetti, Margaret-Ann Bevan (St Thomas' Hospital, London), Sinead McKernan (Royal Victoria Hospital, Belfast), Emily Wandolo (King's College Hospital, London), Celia Richardson,

Elaney Youssef (Brighton and Sussex University Hospital), Pippa Green (Withington Hospital, Manchester), Sue Faulkner (Gloucester Royal Hospital), Rebecca Faville (Whittall Street Clinic, Birmingham), Sandra Herman, Christine Care (Royal Hallamshire Hospital, Sheffield), Helen Blackman (St Mary's Hospital, Portsmouth), and Katharine Bellenger, Keith Fairbrother (Medical Research Council Clinical Trials Unit at UCL, London).

Members of the UK Register Steering Committee: Andrew Phillips (Chair), University College London (UCL), London; Abdel Babiker, UCL, London; Valerie Delpech, Public Health England, London; Sarah Fidler, St. Mary's Hospital, London; Mindy Clarke, Brighton & Sussex University Hospitals NHS Trust, Brighton; Julie Fox, Guys and St Thomas' NHS Trust/Kings College, London; Richard Gilson, West London Centre for Sexual Health, London; David Goldberg, Health Protection Scotland, Glasgow; David Hawkins, Chelsea & Westminster NHS Trust, London; Anne Johnson, UCL, London; Margaret Johnson, UCL and Royal Free NHS Trust, London; Ken McLean, West London Centre for Sexual Health, London; Eleni Nastouli, UCL, London; Frank Post, King's College, London.

The members of the UK Register of HIV seroconverters are: N Kennedy, Monklands Hospital, Airdrie; J Pritchard, Ashford Hospital, Ashford; U Andrady, Ysbyty Gwynedd, Bangor; N Rajda, North Hampshire Hospital, Basingstoke; C Donnelly, S McKernan, Royal Victoria Hospital, Belfast; S Drake, G Gilleran, D White, Birmingham Heartlands Hospital, Birmingham; J Ross, J Harding, R Faville, Whittall Street Clinic, Birmingham; J Sweeney, P Flegg, S Toomer, Blackpool Victoria Hospital, Blackpool; H Wilding, R Woodward, Royal Bournemouth Hospital, Bournemouth; G Dean, C Richardson, N Perry, Royal Sussex County Hospital, Brighton; M Gompels, L Jennings, Southmead Hospital, Bristol; D Bansaal, Queen's Hospital, Burton-Upon-Trent; M Browing, L Connolly, Cardiff Royal Infirmary, Cardiff; B Stanley, North Cumbria Acute Hospitals NHS Trust, Carlisle; S Estreich, A Magdy, St. Helier Hospital, Carshalton; CO'Mahony, Countess of Chester Hospital, Chester; P Fraser, Chesterfield & North Derbyshire Royal Hospital, Chesterfield; SPR Jebakumar, Essex County Hospital, Colchester; L David, Coventry & Warwickshire Hospital, Coventry; R Mette, Mayday University Hospital, Croydon; H Summerfield, Weymouth Community Hospital, Dorset; M Evans, Ninewells Hospital, Dundee; C White, University Hospital of North Durham, Durham; R Robertson, Muirhouse Medical Group, Edinburgh; C Lean, S Morris, Western General Hospital, Edinburgh; A Winter, Gartnavel General Hospital & Glasgow Royal Infirmary, Glasgow; S Faulkner, Gloucestershire Royal Hospital, Gloucester; B Goorney, Salford Hope Hospital, Greater Manchester; L Howard, Farnham Road Hospital, Guildford; I Fairley, C Stemp, Harrogate Hospital, Harrogate; L Short, Huddersfield Royal Infirmary, Huddersfield; M gomez, F young, St Mary's Hospital Isle of Wight; M Roberts, S Green, Kidderminster General Hospital, Kidderminster; K Sivakumar, the Queen Elizabeth Hospital, King's Lynn; J Minton, A Siminoni, Leeds General Infirmary, Leeds; J Calderwood, D Greenhough, J Minton, St. James' Hospital, Leeds; C DeSouza, Lisa Muthern, C Orkin, Barts & the London NHS Trust, London; S Murphy, M Truvedi, Central Middlesex Hospital, London; K McLean, Charing Cross Hospital, London; D Hawkins, C Higgs, A Moyes, Chelsea & Westminster Hospital, London; S Antonucci, S McCormack, Dean Street Clinic, London; W Lynn, Ealing Hospital, London; M Bevan, J Fox, A Teague, Guy's & St. Thomas NHS Trust, London; J Anderson, S Mguni, Homerton Hospital, London; F Post, L Campbell, E Wandolo King's College Hospital, London; C Mazhude, H Russell, Lewisham University Hospital, London; R Gilson, G Carrick, C Young Mortimer Market Centre, London; J Ainsworth, A Waters, North Middlesex Hospital, London; P Byrne, M Johnson, Royal Free Hospital, London; London; S Fidler, K Kuldanek, S Mullaney, St. Mary's Hospital, London; V Lawlor, R Melville, Whipps Cross Hospital, London; A Sukthankar, S Thorpe, Manchester Royal Infirmary, Manchester; C Murphy, E Wilkins, North Manchester General Hospital, Manchester; S Ahmad, P Green, Withington Hospital, Manchester; S Tayal, James Cook Hospital, Middlesbrough; E Ong, Newcastle General Hospital, Newcastle; J Meaden, Norfolk & Norwich University Hospital, Norwich; L Riddell, City Hospital, Nottingham; D Loay, K Peacock, George Eliot Hospital, Nuneaton; H Blackman, V Harindra, St. Mary's Hospital, Portsmouth; AM Saeed, Royal Preston Hospital, Preston; S Allen, U Natarajan, East Surrey Hospital, Redhill; O Williams, Glan Clwyd

District General, Rhyl; H Lacey, Baillie Street Health Centre, Rochdale; C Care, C Bowman, S Herman, Royal Hallamshire Hospital, Sheffield; SV Devendra, J Wither, Royal Shrewsbury Hospital, Shrewsbury; A Bridgwood, G Singh, North Staffordshire Hospital, Stoke-on-Trent; S Bushby, Sunderland Royal Hospital, Sunderland; D Kellock, S Young, King's Mill Centre, Sutton-in-Ashfield; G Rooney, B Snart, the Great Western Hospital, Swindon; J Currie, M. Fitzgerald, Taunton & Somerset Hospital, Taunton; J Arumainayyagam, S Chandramani, Manor Hospital, Walsall; S Rajamanoharan, T Robinson, Watford General Hospital, Watford; M Roberts, Worcester Royal Infirmary, Worcester; O Williams, Maelor Hospital, Wrexham; B Taylor, Wycombe General Hospital, Wycombe; C Brewer, I Fairley, Monkgate Health Centre, York Hospital NHS Trust, York.

HIV-1 Seroconverter Study (Germany):

We would like to thank all members of the German HIV-1 Seroconverter Study Group who participated in this study: **Berlin**: Dres. Mayr, Schmidt, Speidel and Strohbach (Medizinisches Versorgungszentrum, Ärzteforum Seestraße), PD Dr. Arastéh (Auguste-Viktoria-Krankenhaus/ Vivantes), Dr. Cordes, Dres. Stündel and Claus, Dres. Baumgarten, Carganico, Ingiliz and Dupke, Dres. Freiwald and Rausch, Dres. Moll and Schleehauf, Dres. Hintsche and Klausen, Dres. Jessen and Jessen, Dres. Köppe and Kreckel, Dres. Schranz and Fischer, Dres. Schulbin and Speer, Dres. Glaunsinger and Wicke, Dres. Bieniek and Hillenbrand, Dres. Schlote, Lauenroth-Mai and Schuler, Dres. Schürmann and Wesselman (Charité Berlin); **Bochum**: Prof. Dr. Brockmeyer (St. Joseph-Hospital); **Dortmund**: Prof. Dr. Gehring and Dr. Schmalöer and Dr. Hower (Klinikum Dortmund); **Dresden**: Dr. Spornraft-Ragaller (Universitätsklinikum Dresden); **Düsseldorf**: Prof. Dr. Häussinger and PD Dr. Reuter (Universitätsklinik Düsseldorf); **Essen**: Dr. Esser (Universitätsklinikum Essen); **Frankfurt/Oder**: Dr. Markus; **Halle/Saale**: Dr. Kreft (Universitätsklinik Martin-Luther-Universität); **Hamburg**: Dres. Berzow, Christl and Meyer, Prof. Dr. Plettenberg, Dr. Stoehr, Dr. Graefe and Dr. Lorenzen (Institut für Infektionsmedizin, ifi, Allgemeines Krankenhaus St. Georg); Dres. Adam, Schewe and Weitner, Dr. Fenske, Dr.Hansen, Prof. Dr. Stellbrink (Infektionsmedizinisches Zentrum Hamburg, ICH); Dr. Wiemer (Bundeswehrkrankenhaus Hamburg); Dr. Hertling (Universitätsklinikum Hamburg Eppendorf); **Hannover**: Prof. Dr. Schmidt (Medizinische Hochschule Hannover); **Krefeld**: Dr. Arbter; **Ludwigshafen**: Dr. Claus (Klinikum Ludwigshafen); **Mainz**: Prof. Dr. Galle (Klinikum der Joh.-Gutenberg-Universität); **München**: Dres. Jäger and Jägel-Guedes, Dr. Postel, Prof. Dr. Fröschl and Dr. Spinner (Technische Universität München); Prof. Dr. Bogner (Klinikum der Ludwig-Maximilians-Universität); **Regensburg**: Prof. Dr. Salzberger, Prof. Dr. Schölmerich and Dr. Audebert (Universitätsklinik Regensburg); **Salzgitter**: Dr. Marquardt (Klinikum Salzgitter); **Stuttgart**: Dres. Schaffert, Schnaitmann and Trein, Dres. Frietsch, Müller and Ulmer; **Trier**: Dr. Detering-Hübner (Gesundheitsamt Trier); **Ulm**: Prof. Dr. Kern and Prof. Dr. Dr. Kreidler (Universitätsklinik Ulm); **Weil/Rhein**: Dres. Schubert, Dehn and Schreiber; **Wiesbaden**: Dr. Güler. **Robert Koch Institute Berlin**: Dr. Barbara Gunsenheimer-Bartmeyer, MSc. Daniel Schmidt, Dr. Karolin Meixenberger, Prof. Dr. Norbert Bannert.

References for Supplementary Information

1. Sankoff, D. Minimal Mutation Trees of Sequences. *SIAM Journal on Applied Mathematics* **28,** 35–42 (1975).
2. Erdős, P. L. & Székely, L. A. On weighted multiway cuts in trees. *Mathematical Programming* **65,** 93–105 (1994).
